# Interferon-alpha-2b induces autophagy in hepatocellular carcinoma cells through Beclin1 pathway

**DOI:** 10.7497/j.issn.2095-3941.2014.01.006

**Published:** 2014-03

**Authors:** Jun Zhao, Ming-Li Wang, Zeng Li, Dong-Mei Gao, Yu Cai, Jun Chang, Shi-Ping Wang

**Affiliations:** ^1^Department of Parasitology, Xiangya Medical School, Central South University, Changsha 410078, China; ^2^Department of Microbiology, Anhui Medical University, Hefei 230032, China; ^3^Department of Clinical Laboratory, the Third Affiliated Hospital of Anhui Medical University, Hefei 230032, China; ^4^Department of Orthopedics, The First Affiliated Hospital of Anhui Medical University, Hefei 230032, China

**Keywords:** Interferon-alpha-2b (IFN-α2b), autophagy, acridine orange, Beclin1, transmission electron microscopy

## Abstract

**Objective:**

To determine whether Interferon-alpha-2b (IFN-α2b) can modulate the autophagic response in hepatocellular carcinoma cells.

**Methods:**

Hepatocellular carcinoma cells were treated with IFN-α2b. Autophagy was assessed by acridine orange staining, GFP-LC3 dotted assay, transmission electron microscopy and immunoblotting.

**Results:**

Acridine orange staining showed that IFN-α2b triggered the accumulation of acidic vesicular and autolysosomes in HepG2 cells. The acridine orange HepG2 cell ratios were (4.3±1.0)%, (6.9±1.4)%, and (13.1±2.3)%, respectively, after treatment with 100, 1,000, and 10,000 IU/mL IFN-α2b for 48 h. A markedly punctate pattern was observed in HepG2 cells treated with 10,000 IU/mL IFN-α2b for 48 h, but only diffuse and weakly fluorescent GFP-LC3 puncta was observed in control cells. HepG2 cells treated with 10,000 IU/mL IFN-α2b for 48 h developed autophagosome-like characteristics, including single- or double-membrane vacuoles containing intact and degraded cellular debris. The Beclin1 and LC3-II protein expression was up-regulated by IFN-α2b treatment.

**Conclusion:**

Autophagy can be induced in a dose-dependent manner by treatment with IFN-α2b in HepG2 cells, and the Beclin1 signaling pathway was stimulated by IFN-α2b.

## Introduction

Interferon-alpha (IFN-α) is cytokines belonging to the type I IFN group. They exert many effects on cell functions^1^^,^^2^. The IFN-α family contains at least 13 functional IFN subtypes, all of which share the same receptor system and exert similar biological activities^3^. Study of 50 years on IFN-α has revealed that these cytokines exhibit a variety of biological effects which are different from those on viral replication, such as antitumor activity. IFN-α cytokines are widely expressed and secreted as the first line of defense against several types of tumors. They have been used in over 40 countries for the treatment of more than 14 types of cancer, including some hematological malignancies (hairy cell leukemia, chronic myeloid leukemia, some B- and T-cell lymphomas) and certain solid tumors, such as melanoma, renal carcinoma and Kaposi’s sarcoma. IFN-α can directly inhibit the proliferation of normal and tumor cells *in vitro* and *in vivo*, and can exert other direct effects on tumor cells^4^^-^^6^.

In the present study we investigated the effect of IFN-α2b on autophagy in hepatocellular carcinoma cells and related mechanisms.

## Materials and methods

### Cell culture

Hepatocellular carcinoma HepG2 cell line was procured from American type culture collection (ATCC). We propagated HepG2 cells in Dulbecco’s modified Eagle’s medium (GBICO) supplemented with 10% fetal bovine serum (GBICO) in a humidified incubator containing 5% CO_2_ at 37 °C. Human IFN-α2b (Sigma-Aldrich) was diluted in serum-free medium.

### Acridine orange staining for autophagy

Autophagy is characterized by the formation and promotion of acidic vesicular organelles (AVOs). HepG2 Cells were treated with various concentrations of IFN-α2b in 6-well plates. After 48 h post treatment, then incubated with 1 mg/mL acridine orange (Sigma) for 15 min. Pictures were obtained with a fluorescence microscope.

### GFP-LC3 dotted assay

Cells were transiently transfected with GFP-LC3 (Origene Tech. Inc, MD, USA) vector using Lipofectamine LTX and PLUS Reagents (Invitrogen Corporation) according to the manufacturer’s instructions. After 24 h, the cells were exposed to IFN-α2b for 48 h as indicated, and examined under fluorescence microscope. The induction of autophagy was quantified by counting the percentage of cells in each group with a number of LC3 aggregates.

### Transmission electron microscopy

Cells were fixed with 3% glutaraldehyde in 0.1 M cacodylate buffer for 1 h. After fixation, the samples were post-fixed in 1% OsO_4_ in the same buffer for 30 min. Ultrathin sections were then observed under a transmission electron microscope.

### Western blot

Western blotting procedure was performed as described previously^7^. Briefly, cells were lysed in appropriate volume of lysis buffer (Sigma Aldrich). Fifty micrograms of protein samples were separated by SDS-PAGE and transferred onto nitrocellulose membrane. The membranes were immunoblotted with primary antibodies purchased from Santa Cruz Biotechnology, Inc. (Santa Cruz, CA, USA). Blots were incubated with horseradish peroxide-conjugated goat anti-rabbit or goat anti mouse secondary antibodies purchased from Santa Cruz Biotechnology. All experiments were performed and verified using at least three biological replicates.

### Statistical analysis

The experimental data were expressed as mean ± SD. Group means were compared by *t*-test using the statistical software program SPSS 13.0. *P* values <0.05 were considered to be statistically significant.

## Results

**Figure 1:**
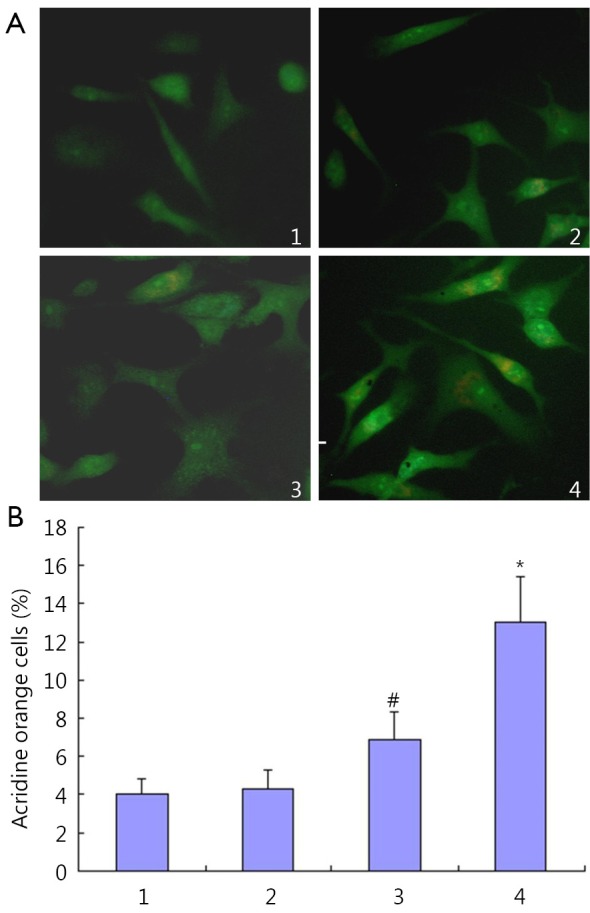
Modulation of autophagy by IFN-α2b in HepG2 cells. Cells were treated with IFN-α2b for 48 h at concentrations of 100, 1,000, and 10,000 IU/mL. Cells were then stained with acridine orange. 1, control group; 2, cells treated with 100 IU/mL IFN-α2b; 3, cells treated with 1,000 IU/mL IFN-α2b; 4, cells treated with 10,000 IU/mL IFN-α2b. (A) autophagic vacuoles were observed and imaged on a fluorescence microscope; (B) acridine orange staining positive cells (%). 1, control group; 2, cells treated with 100 IU/mL IFN-α2b; 3, cells treated with 1,000 IU/mL IFN-α2b; 4, cells treated with 10,000 IU/mL IFN-α2b (#*P*<0.05 relative to control values and *P<0.01 relative to control values).

HepG2 cells were treated with IFN-α2b. IFN-α2b was found to trigger the accumulation of acidic vesicular and autolysosomes in HepG2 cells (**Figure 1A**). The acridine orange HepG2 cell ratios were (4.3±1.0)%, (6.9±1.4)%, and (13.1±2.3)% after treatment with 100, 1,000, and 10,000 IU/mL IFN-α2b, respectively (**Figure 1B**).

**Figure 2:**
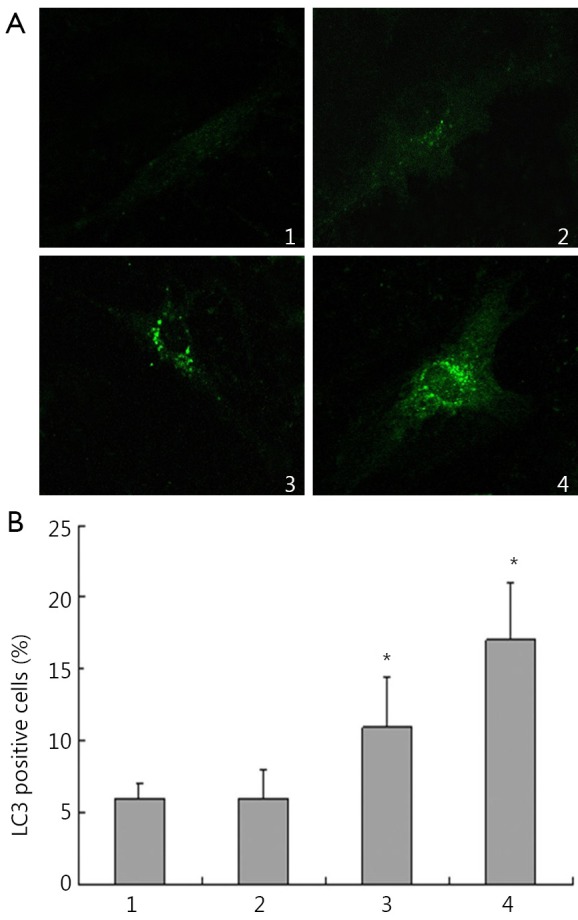
IFN-α2b induced punctuation of GFP-LC3 distribution in HepG2 cells. At 24 h after the transient transfection of GFP-LC3, cells were treated with IFN-α2b for 48 h and then analyzed for fluorescence. A. Images were captured using a fluorescence microscope. 1, control group; 2, cells treated with 100 IU/mL IFN-α2b; 3, cells treated with 1,000 IU/mL IFN-α2b; 4, cells treated with 10,000 IU/mL IFN-α2b; B. LC3 positive cells (%). 1, control group; 2, cells treated with 100 IU/mL IFN-α2b; 3, cells treated with 1,000 IU/mL IFN-α2b; 4, cells treated with 10,000 IU/mL IFN-α2b (*P<0.01 relative to control values).

**Figure 3:**
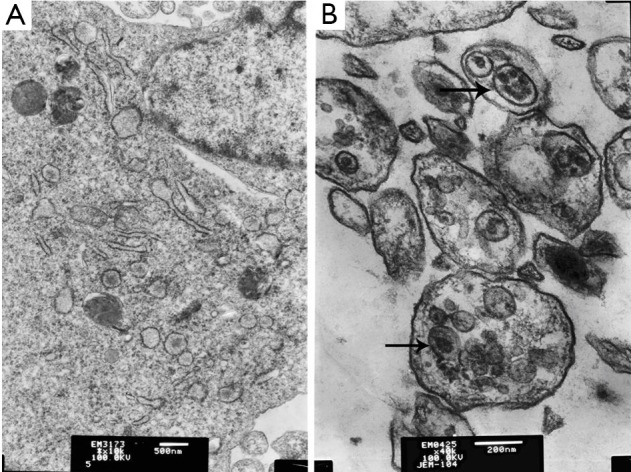
Transmission electron images of HepG2 cells treated with 10,000 IU/mL IFN-α2b. The arrowhead indicates single- or double-membrane vesicles containing intact and degraded cellular debris. (A) Control; (B) Cells treated with 10,000 IU/mL IFN-α2b.

GFP-LC3 plasmid was transfected into HepG2 cells for observation and quantification of the redistribution of autophagy marker LC3 from a diffused to punctate pattern after treatment with IFN-α2b for 48 h. Similarly, as shown in **Figure 2**, a markedly punctate pattern appeared among HepG2 cells treated with 10,000 IU/mL IFN-α2b for 48 h, but there was only diffuse and weak fluorescent GFP-LC3 puncta among control cells. HepG2 cells treated with 10,000 IU/mL IFN-α2b for 48 h developed autophagosome-like characteristics, including single- or double-membrane vacuoles containing intact and degraded cellular debris (**Figure 3**).

**Figure 4:**
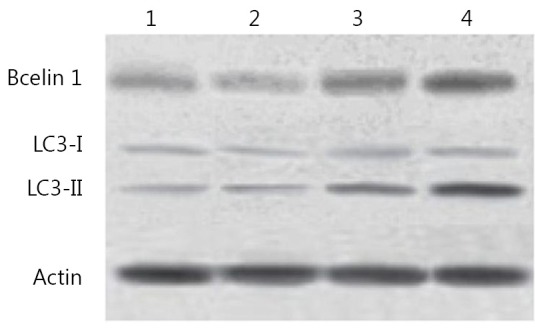
Modification of LC3 and Bcelin1 in HepG2 cells after treatment with IFN-α2b. 1, control group; 2, cells treated with 100 IU/mL IFN-α2b; 3, cells treated with 1,000 IU/mL IFN-α2b; 4, cells treated with 10,000 IU/mL IFN-α2b.

The autophagy in HepG2 cells was also confirmed by immunoblotting, which showed the degree of accumulation of LC3 to be correlated with the number of autophagosomes relative to the amount of endogenous LC3-II protein. Consistent with the data obtained from GFP-LC3-transfected cells, Western blot recorded a strong increase in the amount of endogenous LC3-II in HepG2 cells after treatment with 10,000 IU/mL IFN-α2b for 48 h (**Figure 4**). The molecular mechanism underlying autophagy induction was determined using IFN-α2b. The protein expression of Beclin1 was found to be up-regulated by IFN-α2b. These results indicated that IFN-α2b induced HepG2 cell autophagy exerted its effects through the Beclin1 pathway.

## Discussion

Hepatocellular carcinoma is the fifth most common cancer in the world. However, the potentially curable method is only possible for a small proportion of those afflicted, for the rest, palliative treatment is indicated. In this setting, type I IFN has emerged as an alternative treatment modality for hepatocellular carcinoma^8^^,^^9^.

Several biological functions of type I IFN, including its regulation of innate and adaptive immunity and its antiangiogenic and proapoptotic effects, make it an obvious candidate for anti-cancer therapy. Indeed, type I IFN has been used with some success for the treatment of several types of cancer, including hematological malignancies and solid tumors^10^. It was recently shown that IFN-α2c could induce autophagy in HeLa S3, MDA-MB-231, T98G and A549 cell lines^11^. But IFN-α2c is rarely used in the clinical treatment of cancer, and IFN-α2b is the main drug treatment for cancer.

Autophagy is a self-degradation process whereby cytosolic components and organelles are sequestered in double membrane-bound vesicles and delivered to lysosomes for degradation and recycling. In normal tissue, autophagy maintains cellular homeostasis by clearing damaged organelles or misfolded proteins. However, the role of autophagy in cancer is complex and paradoxical as it is an adaptive process that is responsive to changes in the cellular microenvironment. Thus, autophagy can either suppress or support the growth of tumor cells depending on the cellular context^12^.

Given the critical roles of autophagy in tumor progression and maintenance, various preclinical and clinical studies have been undertaken to develop therapeutic agents targeting autophagy. As most anticancer agents inevitably cause cellular stress, autophagy is often activated in cancer cells after drug treatment. Indeed, many therapies targeting growth factor signaling-either singly or in combinations targeting two different pathways-lead to autophagy induction. For example, several allosteric and catalytic inhibitors of mTOR, PI3K-AKT, and the tyrosine kinase signaling and activators of energy sensing pathway induce autophagy in cells. It was originally proposed that autophagic cell death is part of the mechanism of action of anticancer drugs^13^. Beclin 1, which was the first gene to be identified positively associated with mammalian autophagy, plays a central role in coordinating the cytoprotective function of autophagy and in opposing apoptosis^14^.

In this study, hepatocellular carcinoma cells were treated with IFN-α2b. Autophagy was assessed by acridine orange staining, GFP-LC3 dotted assay, transmission electron microscopy and immunoblotting. We found that autophagy can be induced in a dose-dependent manner by treatment with IFN-α2b in HepG2 cells. The dependence of IFN-α2b induced autophagy on the Beclin1 pathway was also assessed, and the Beclin1 signaling pathway was stimulated by IFN-α2b.
